# Colour compound lenses for a portable fluorescence microscope

**DOI:** 10.1038/s41377-019-0187-1

**Published:** 2019-08-21

**Authors:** Bo Dai, Ziao Jiao, Lulu Zheng, Hunter Bachman, Yongfeng Fu, Xinjun Wan, Yule Zhang, Yu Huang, Xiaodian Han, Chenglong Zhao, Tony Jun Huang, Songlin Zhuang, Dawei Zhang

**Affiliations:** 1 Engineering Research Center of Optical Instrument and System, the Ministry of Education, Shanghai Key Laboratory of Modern Optical System, University of Shanghai for Science and Technology, 200093 Shanghai, China; 2 Department of Mechanical Engineering and Materials Science, Duke University, Durham, NC 27709 USA; 3 Department of Medical Microbiology and Parasitology, School of Basic Medical Sciences, Fudan University, 200032 Shanghai, China; 4 Department of Laboratory Medicine, Shanghai Cancer Center, Fudan University, 200032 Shanghai, China; 5 Department of Physics, University of Dayton, Dayton, OH 45469 USA; 6 Department of Electro-Optics and Photonics, University of Dayton, Dayton, OH 45469 USA

**Keywords:** Optics and photonics, Optical materials and structures

## Abstract

In this article, we demonstrated a handheld smartphone fluorescence microscope (HSFM) that integrates dual-functional polymer lenses with a smartphone. The HSFM consists of a smartphone, a field-portable illumination source, and a dual-functional polymer lens that performs both optical imaging and filtering. Therefore, compared with the existing smartphone fluorescence microscope, the HSFM does not need any additional optical filters. Although fluorescence imaging has traditionally played an indispensable role in biomedical and clinical applications due to its high specificity and sensitivity for detecting cells, proteins, DNAs/RNAs, etc., the bulky elements of conventional fluorescence microscopes make them inconvenient for use in point-of-care diagnosis. The HSFM demonstrated in this article solves this problem by providing a multifunctional, miniature, small-form-factor fluorescence module. This multifunctional fluorescence module can be seamlessly attached to any smartphone camera for both bright-field and fluorescence imaging at cellular-scale resolutions without the use of additional bulky lenses/filters; in fact, the HSFM achieves magnification and light filtration using a single lens. Cell and tissue observation, cell counting, plasmid transfection evaluation, and superoxide production analysis were performed using this device. Notably, this lens system has the unique capability of functioning with numerous smartphones, irrespective of the smartphone model and the camera technology housed within each device. As such, this HSFM has the potential to pave the way for real-time point-of-care diagnosis and opens up countless possibilities for personalized medicine.

## Fluorescence microscopy: Smartphone solutions for seeing cells

A method of turning a smartphone into a fluorescence microscope, developed by researchers in the US and China, enables complex biomedical analyses to be performed rapidly and inexpensively. Conventional fluorescence microscopes are vital for detecting specific cell types and proteins, but are bulky and inconvenient for point-of-care diagnoses. Tony Jun Huang at Duke University, Dawei Zhang at USST and co-workers used liquid polymers to create miniature lenses comprising two droplets, one inside the other, dyed with colored solvents. The lenses, which are compatible with several different smartphone cameras, allowed the researchers not only to observe and count cells, but also to monitor the expression of fluorescently-tagged genes, and to distinguish between normal tissue and tumors. This ingenious use of easily-accessible and affordable smartphone technology will lead to better on-site personalized medicine, especially for developing countries.

## Introduction

Fluorescence microscopy is ubiquitous in applications ranging from biological research^1–5^ and healthcare^6,7^ to environmental monitoring^8,9^ and food sanitation^10,11^. In the fields of biomedical study and clinical applications, fluorescence imaging allows the detection and tracking of cells, proteins, and other molecules of interest in a specimen with high sensitivity and precision^12–18^. A conventional fluorescence microscope is typically built from a series of bulky elements including free-space optics, high-cost image sensors, heavy mechanical components, and a stand-alone computer for data analysis. The bulky and high-cost nature of these conventional fluorescence microscopes make them extremely challenging for use in point-of-care diagnosis, especially in resource-limited areas^19^. Therefore, the development of portable fluorescence microscopes is extremely important for point-of-care diagnosis and personalized medicine. The smartphone is an ideal platform for this purpose because of its portability and accessibility to a broad range of users^20–25^. In addition, the boom in smartphone-based technology over the last several decades has provided smartphones with the power to replace traditional microscopes; the integrated modules of a smartphone can readily serve as sensors, portable computation units, information centres, or stand-alone devices for customized applications within fluorescence microscopy.

Based on these unique features, several smartphone-based microscopes have been demonstrated and are attracting increasing interest^26–32^. For example, fluorescence microscopy imaging of human blood cells, waterborne parasites, and human cytomegaloviruses has been realized by using a smartphone^33–37^. Throughout these research efforts, the key elements for a smartphone-based fluorescence microscope, such as light-emitting diodes (LED) for illumination, external lenses for optical imaging and proper magnification, and fluorescence emission filters for routing light, have been developed. As a low-cost solution, a ball lens was demonstrated as an external lens to be used in conjunction with a smartphone camera to form a microscope^38,39^. Since polymer lenses are easy to produce and can provide high resolving power, they are very suitable for developing a do-it-yourself microscope for some resource-limited applications^31,40–42^. Because of the small pixel size (<1.5 μm) of smartphone image sensors, a lens with unit optical magnification has proven good enough for cell detection^36,43^. Thin-film Fabry–Perot interference filters have been used as optical filters to achieve fluorescence imaging with a high signal-to-noise ratio at an affordable cost. Alternatively, if the propagation of the excitation light is not against the detection path, inexpensive plastic colour filters are also acceptable for creating dark-field backgrounds^33^.

Several smartphone-based diagnostic platforms have been developed by assembling all of these elements (LEDs, additional lenses, and necessary filters) into standalone attachments for a smartphone^44–46^. These attachments are traditionally tailored to the specific hardware of the smartphone model that they are attached to; however, due to the ever-changing technology of mobile phones, an attachment whose design is independent from a specific phone model is preferred. To address this problem, we developed a low-cost handheld smartphone fluorescence microscopy (HSFM) that can achieve all of the functions of a conventional fluorescence microscopy yet still has a field-portable size. The key element of our HSFM is that it uses a single compact and multifunctional colour lens to turn any smartphone model into a fluorescence microscope without needing to modify the design of the attachment when switching phones. This capability means that our device achieves magnified fluorescent imaging using a single lens without traditional filters. This significantly reduces the complexity of the design, while simultaneously allowing it to be adopted onto a variety of smartphone designs. The unique features of our HSFM are as follows: (1) consistent function independent of phone model; (2) small form factor; (3) ease of operation; (4) low cost; and (5) ability to be mass produced. Bright-field and fluorescence imaging within three typical fluorescence channels was demonstrated in several bioanalytical applications, including cell and tissue observation, cell counting, plasmid transfection evaluation, and superoxide production analysis.

## Results

### Fabrication of colour compound lenses

**Fig. 1:**
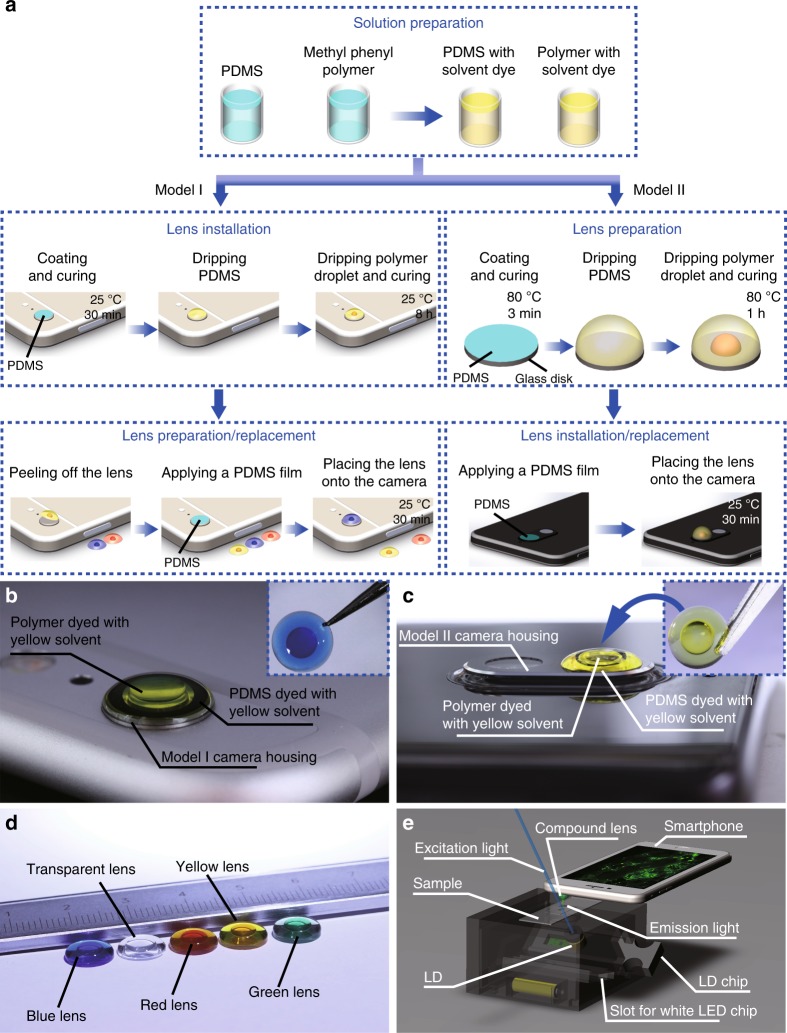
Fabrication process of the colour compound lens. **a** Fabrication process for constructing colour compound lenses for smartphones with round protruding camera housings, as well as less accessible camera housings. The colour compound lenses for phones without protruding lenses are prepared on a stand-alone glass disk for future placement on the camera lens. **b** A yellow lens is directly fabricated on the smartphone that has a round protruding camera housing (Model I). Inset: the preprepared blue lens peeled off from the camera housing. **c** A yellow lens is transferred onto a smartphone with the other camera housing type (Model II). Inset: the yellow lens for installation onto the camera housing. **d** Blue, transparent, red, yellow, and green lenses were fabricated on glass disks to create various fluorescence filters. **e** Schematic diagram of fluorescence imaging. The smartphone equipped with a green lens is to capture green fluorescence from a sample illuminated by a blue light beam

The fluorescent module of our HSFM consists of a colour compound lens that achieves both imaging and light filtering. The miniature lens is made of two high-refractive-index droplets, one inside the other, which are dyed with coloured solvents that only transmit the desired emission light to the imaging sensor. The fabrication process for the colour compound lenses is illustrated in Fig. 1a; the fabrication process varies based on the design of a smartphone’s camera lens—either the lens protrudes from the back of the phone (hereinafter called Model I), or it is more in profile with the remainder of the phone (or simply Model II). In both lens designs, coloured polydimethylsiloxane (PDMS) prepolymer and methyl phenyl polymer (vinyl-terminated dimethyl diphenyl polysiloxanes) are first prepared by dissolving solvent dyes in the liquids. Then, PDMS cross-linker is mixed with the prepolymer at a 1:5 weight ratio. If the camera housing is round and protrudes directly from the back of the phone, a colour lens can be fabricated easily by successively dripping the PDMS (density: 965 kg m^–3^, refractive index: 1.434) and the polymer (density: 1100 kg m^–3^, refractive index: 1.540) on the camera housing, which has had a thin PDMS film deposited on its surface in preparation. After 8 h of curing the PDMS at room temperature, a two-droplet lens is formed on the round protruding camera housing, as shown in Fig. 1b. The high-refractive-index polymer droplet remains in the liquid state, and the solidified elastic PDMS encapsulates it. Alternatively, if the lens does not protrude accessibly from the rear of the phone, then the two-droplet lens can first be formed on a glass disk that is coated with a thin PDMS film and then transferred onto the camera housing; the camera lens also has a thin layer of PDMS deposited on it so that we can properly secure the lens. Figure 1c, d show lenses that were formed on a separate disk and then transferred to the camera housing. The different lenses fabricated for smartphones with the Model I or Model II camera housings are shown in Fig. S1 in the Supplementary Information.

During the fabrication process, after dripping the liquid PDMS onto either the round protruding camera housing or the glass disk, the radius of the droplet and the capillary length determine how the droplet spreads. If the radius of the PDMS droplet is larger than the capillary length, $\ell _c = \sqrt {{\mathrm{\gamma }}/\left( {\rho g} \right)}$, where *γ* is the liquid-vapour surface tension, *ρ* is the density of the droplet, and *g* is the acceleration due to gravity, then the gravitational force plays a dominant role in driving the droplet to axisymmetrically spread in a stick-slip motion. Once the three-phase contact line (the liquid PDMS, air, and glass) reaches the edge of the substrate, the PDMS naturally stops spreading and begins to bulge into a spherical cap; this behaviour is why a lens needs to be fabricated on a disk and then transferred onto the phone for camera housings that are not circular and protruding from the surface of the phone. After dripping the PDMS onto the substrate, we deposit the polymer droplet onto the centre of the PDMS, where it sinks to the bottom inside the PDMS. Due to the additional volume of the submerged polymer droplet, the surface of the PDMS spherical cap becomes more curved, and the contact angle of the PDMS at the edge concomitantly increases. The PDMS droplet is stable on the substrate and will not flow across the edge as long as the contact angle, *θ*_PDMS_, is smaller than the critical angle *θ*_C_ for spreading over the edge; the critical angle is defined by the Gibbs inequality equation as *θ*_C_ = (180°–*φ*) + *θ*_e_, where *φ* is the subtended angle at the edge of the camera housing or the glass disk and *θ*_e_ is the thermodynamic equilibrium contact angle^47,48^. *θ*_e_ is 20 ± 0.8° for the PDMS droplet on the cured PDMS film.

### Characteristics of colour compound lenses

**Fig. 2:**
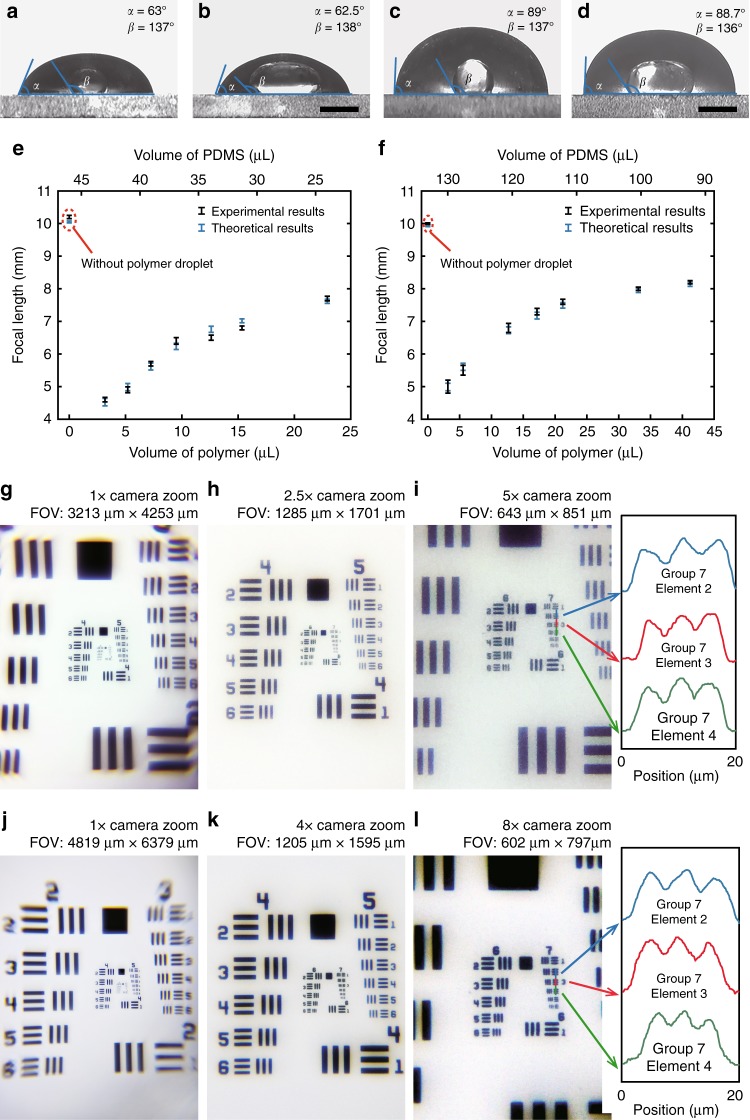
Characterization of the colour compound lens. **a**, **b** Measured contact angles for the Model I camera housing with polymer volumes of 9.5 and 22.9 μL. Scale bar = 2 mm. **c**, **d** Measured contact angles for the Model II camera housing, where the polymer volume was 12.7 and 21.2 μL. Scale bar = 2 mm. Focal length as a function of the polymer and PDMS volumes for the camera housing of **f** Model I and **e** Model II, respectively. Images of the resolution target USAF-1951 with different camera magnifications captured by the camera in **g**–**i** Model I and **j**–**l** Model II housing. The right insets show the intensity profiles along the blue, red, and green lines

In the equilibrium state, the PDMS droplet is in the shape of a spherical cap, as shown in Fig. 2a–d, because the interfacial tension force plays a dominant role and directs the droplet into the shape that has the lowest free energy (a sphere). The contact angles of the PDMS on the camera housing and the standalone disk are ∼62.8° ± 0.5° and 88.4° ± 1.3°, respectively. The shape of the polymer droplet within the PDMS is determined by the interplay between the interfacial tension force and gravity. If the polymer droplet is sufficiently small, the interfacial tension force is dominant, and the shape can be regarded as a segment of a sphere. With an increase in the polymer volume, gravity gradually becomes dominant and distorts the droplet into an oblate spheroidal cap. It is worth noting that the polymer droplet should be large enough to have a base diameter, *D*_Oil_, larger than the aperture of the camera lens. Taking these factors into account, an ellipsoidal droplet model was applied in the analysis of these systems^49^. The external curvature of the PDMS cap was determined by fitting with a circle, whereas the internal PDMS/polymer interface curvature was fitted with a quartic polynomial based on the elliptical profile of the polymer droplet (see the model of the compound lens in Fig. S2 in the Supplementary Information). The focal length of the lenses with a diameter of 7 mm was theoretically calculated using ray tracing and experimentally measured in an optical imaging system, as plotted in Fig. 2e, f (for more details, see the ‘Methods' section and Fig. S3 in the Supplementary Information). The focal length changes with the volume of the polymer owing to the variation in the internal PDMS-polymer interface curvature. The ratio of the semi-major and semi-minor axes of the elliptical profile increases with the polymer volume, resulting in a decrease in the curvature (see Fig. S4 in the Supplementary Information). The lenses fabricated on the protruding camera housing and the glass disk have a focal length of 4.5–7.7 mm and 4.9–8.2 mm, respectively. The manufacturing tolerance of the lenses is ∼2% (Model I) and 4% (Model II) in terms of their focal lengths. In comparison with the lenses made of PDMS only (as marked in the red dashed circles), the lenses with the high-refractive-index polymer droplet inside the PDMS cap have shorter focal lengths. Since the effective aperture is fixed to that of the camera module of the smartphone, the lenses of short focal length could contribute to high resolution in microscopic imaging. Figure 2g–l provides bright-field images of the resolution test target captured by smartphones equipped with lenses made of 3.2 μL polymer droplets. The cameras are capable of resolving a 2.76 μm line. The field of views (FOVs) of the images are 30.74 and 13.66 mm^2^. Due to the curvature-field aberration and pincushion distortion, the edges of the images become blurred. The effective FOVs for clearly resolving 2.76 μm lines are 1.2 × 1.2 mm^2^ (Model I) and 1.6 × 1.6 mm^2^ (Model II), in which the distortion is within ±5%. (see Figs. S5 and S6 in the Supplementary Information). With an appropriate digital zoom from the smartphone, details in the centre area can be clearly viewed.

As desired, lenses dyed with different coloured solvents have different spectral responses with regard to optical density (see ‘Methods' section and Fig. S7 in the Supplementary Information). The green lens has a pass band centred at 550 nm. The red and yellow lenses function as longpass filters with cut-on wavelengths of 594 and 562 nm, respectively. The blue lens allows light around ∼440 nm and above 680 nm to pass through. Thus, in fluorescence microscopic imaging, our lenses not only enable image magnification but also selectively allow emission light to pass through.

Furthermore, since the liquid-state polymer droplet is completely sealed inside the stable, cured PDMS spherical cap, problems associated with external mechanical vibrations, thermal disturbances, and chemical deteriorations can be avoided. Once the lens is adhered to the camera housing, it becomes a part of the smartphone and is convenient to be carried around. The lens can be easily peeled off from the camera housing and replaced with another preprepared lens to alter the imaging modality or the fluorescence channel.

### Cell observation and cell counting

**Fig. 3:**
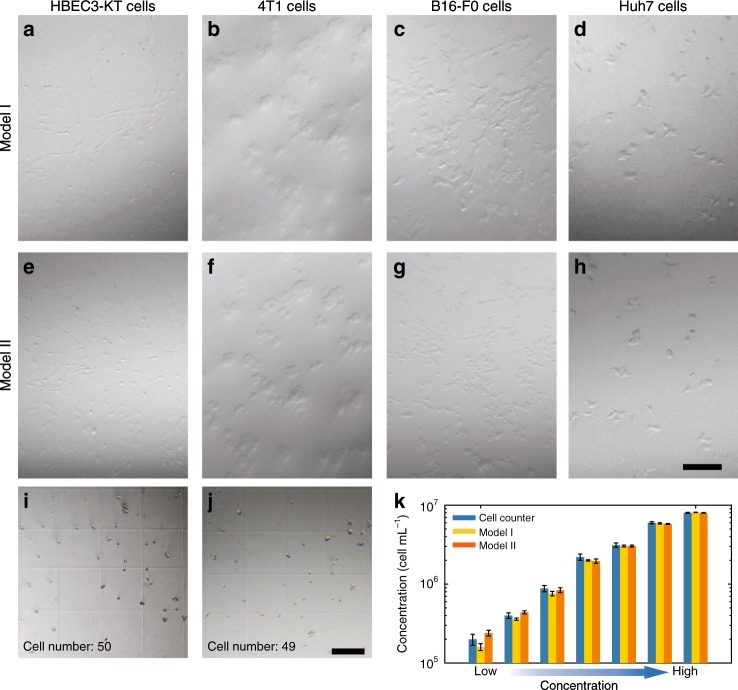
Cell observation and cell counting using HSFM. **a**–**h** Bright-field images of HBEC3-KT cells, 4T1 cells, B16-F0 cells, and Hub7 cells. Scale bar = 100 μm. **i**, **j** Images of A375 cells in a Fuchs-Rosenthal chamber for concentration analysis. Scale bar = 200 μm. **k** Cell counting results obtained by the smartphones and a cell counter

A customized illumination tool was developed and employed in the microscopic imaging process (for more details, see the ‘Methods' section and Fig. S8 in the Supplementary Information). Cells were observed and counted, when exposed to oblique white light illumination. Figure 3a–h show images captured by the smartphone equipped with a transparent lens. The HBEC3-KT cells present epithelial morphology. The cuboidal 4T1 cells and the spindle-shaped B16-F0 cells aggregate in small clusters. The Huh7 cells have a hummingbird phenotype. Zoomed-in bright-field and fluorescence images of 4T1 cells and B16F0 cells can be found in Fig. S9 in the Supplementary Information. In addition, bright-field images and fluorescence images in two fluorescence channels with different optical magnifications of 4T1 cells stained with Hoechst 33342 and calcein acetoxymethyl ester (Calcein-AM) are demonstrated in Fig. S10 in the Supplementary Information. During fluorescence imaging, the excitation light illuminates the sample at an angle larger than the acceptance angle of the lens; this prevents coupling of the excitation and emission light that reaches the image sensor, eliminating background noise (as illustrated in Fig. 1e).

In the cell counting experiment, A375 cells at different concentrations were counted in a Fuchs-Rosenthal chamber. The individual cells can be clearly distinguished, as shown in Fig. 3i, j. The cell concentration was calculated and is depicted in Fig. 3k. The concentration measured by the HSFM exhibits excellent agreement with the results obtained from a commercial cell counter (Countess II, ThermoFisher, USA).

### Immunofluorescence

**Fig. 4:**
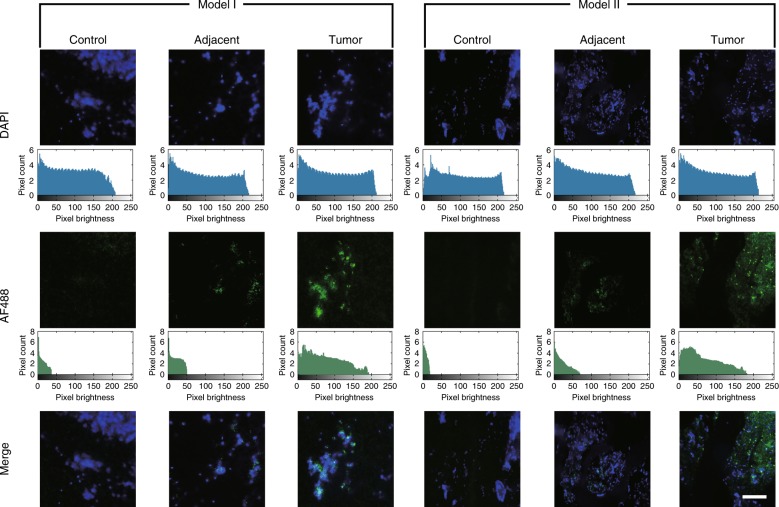
Fluorescence images of human liver tissues using the HSFM. The excitation wavelengths for DAPI and AF488 were 365 and 480 nm, respectively. The images were captured by the smartphone equipped with the blue lens and the green lens. The histogram is in log scale. Scale bars = 50 μm

Human liver tissues incubated with cytokeratine-18 (CK18) antibody and Alexa Fluor 488 (AF488)-conjugated secondary antibody were detected by the smartphone equipped with the green lens. CK18, as a prognostic biomarker, is involved in both cell motility and cancer progression; the positive expression of CK18 is considered suggestive of oncofoetal transformation, malignant transformation, or initiation of abnormal cell differentiation^50,51^. Images of normal tissues, paratumour tissues, and cancer tissues are shown in Fig. 4. The images from different fluorescence channels can be mapped into the same coordinates by image registration before generating composite images (for more details about capturing the images at the same spot, see the Supplementary Note). Image histograms indicate that the settings of the cameras are suitable because the fluorescence emission presents higher brightness than the noise, including dark-current noise and background fluorescence, and no overexposure occurs. A large amount of bright green fluorescence emission in the tumour tissues indicates a high CK18 expression level, confirming a cancer diagnosis. The expression of CK18 in the paratumour tissues was comparatively lower than that in the cancer tissues but was still higher than that in the normal tissues. In addition, a large view of the tissue obtained from a human pancreatic tumour xenograft model in a nude mouse that was injected subcutaneously with B×PC-3 human pancreatic cancer cells was detected (see Fig. S11 in the Supplementary Information). The tissue was stained with a primary rabbit anti-human polyclonal antibody against glyceraldehyde-3-phosphate dehydrogenase (GAPDH), which functions as a housekeeping protein in glycolysis and is associated with tumour development due to the activation of cell proliferation and inflammation^52,53^, and it was also stained with a secondary goat anti-rabbit antibody conjugated with AF488. The tissue sample had a size of 2.8 × 4 mm (length × width), and during detection, almost the entire sample can be clearly visualized.

### Evaluation of plasmid transfection

**Fig. 5:**
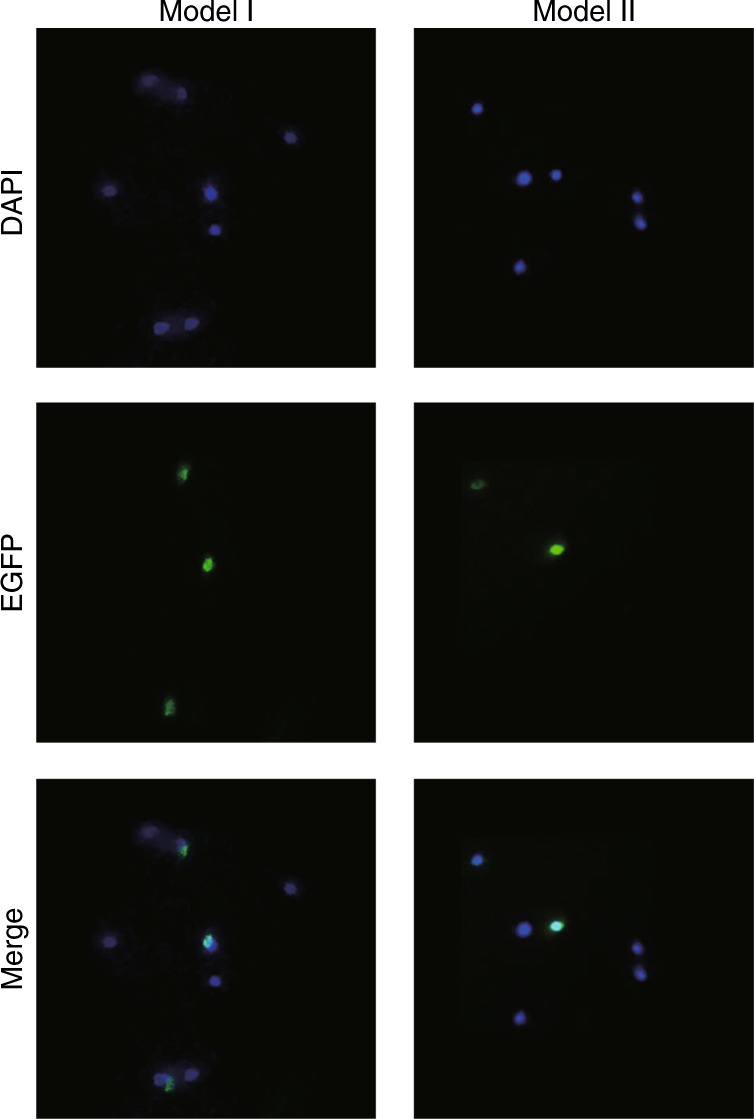
Fluorescence images of the EGFP-tagged human NLRP3 gene in 293T cells using the HSFM. The excitation wavelengths for DAPI and EGFP were 365 and 480 nm, respectively. The images were captured by the smartphone equipped with the blue lens and the green lens. Scale bar = 50 μm

The smartphone equipped with the green lens was employed to monitor the transfection and expression of enhanced green fluorescent protein (EGFP), which is widely used as a reporter gene for the analysis of colocalization and the study of dynamic physiological processes. In the demonstration of plasmid transfection, the EGFP-tagged human NLRP3 gene is transfected into 293T cells. When the transfectant cells are excited by the 480 nm blue light, the EGFP emits bright green fluorescence light at ∼500–550 nm in wavelength. The excitation light is filtered by the green lens, and the fluorescence emission is captured by the smartphone. The green spots in Fig. 5 indicate the expression of EGFP. The transfection efficiency measured 48 h post transfection was calculated to be 38% (Model I) and 33% (Model II). The results are in good agreement with the value measured (34%) from a conventional microscope (Eclipse Ti, Nikon, Japan).

### Quantitative analysis of superoxide production

**Fig. 6:**
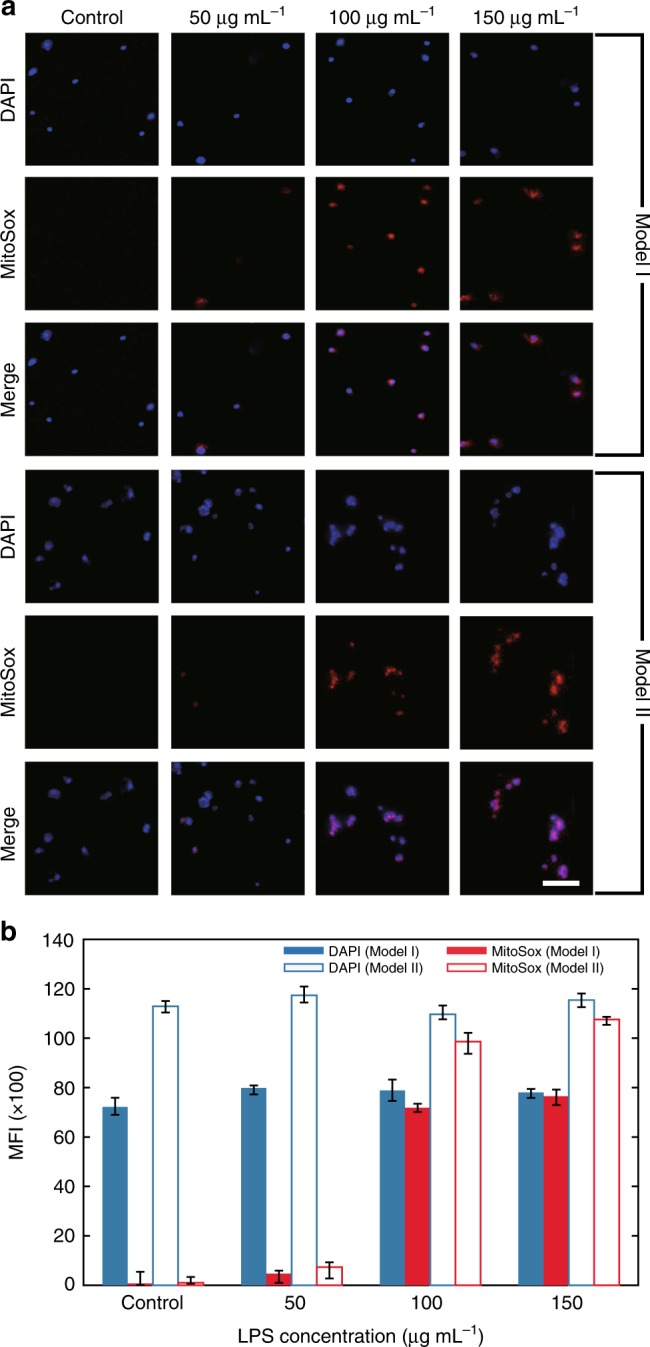
Evaluation of superoxide production using the HSFM. **a** Fluorescence images of LPS-stimulated HBEC3-KT cells stained with DAPI and MitoSOX Red and excited at 365 and 520 nm, respectively. The images were captured by the smartphone equipped with the blue lens and the red lens. Scale bar = 50 μm. **b** Mitochondrial superoxide levels in HBEC3-KT cells exposed to LPS at different concentrations

An increase in cellular superoxide production is associated with cardiovascular diseases and neurodegenerative diseases^54,55^. Lipopolysaccharide (LPS) is capable of priming various cells for the enhanced release of superoxide. MitoSOX Red, a fluorogenic probe with excitation and emission maxima of ∼510 and 580 nm, respectively, is an indicator for the highly selective detection of superoxide. The dose response to LPS stimulation in HBEC3-KT cells was quantitatively analysed. Fluorescence microscopy images of the cells, as shown in Fig. 6a, stained with 4′,6-diamidino-2-phenylindole (DAPI) and MitoSOX Red after exposure to LPS at concentrations of 50, 100, and 150 μg mL^−1^ for 24 h were successively captured by the smartphone equipped with the blue lens and the red lens. Illumination at 365 and 520 nm was used as excitation light for DAPI and MitoSOX Red, respectively. The mean fluorescence intensity (MFI) was calculated, as shown in Fig. 6b. The discrepancy between the results obtained by the two smartphones can be attributed to the difference in sensitivity of the image sensors (for more details, see Fig. S12 in the Supplementary Information). Generally, LPS dose-dependent increases in red fluorescence indicate the enhanced production of superoxides. In comparison with the almost unchanged MFI of DAPI, the consistent increase in the MFI of MitoSOX Red from one phone to the other supports this conclusion.

## Discussion

Compound lenses with various focal lengths can be designed by controlling the volume of the polymer droplet, which varies the droplet profile and subsequent focal length. Short focal lengths ranging from 4.5 to 8 mm can be realized. The optical power, i.e., the reciprocal of the focal length, can be significantly improved by the presence of a high-refractive-index polymer droplet. In addition to optical magnification, the lens dyed with coloured solvents contributes another attractive feature, i.e., light filtering. The PDMS and polymer are transparent over the entire visible band, but the PDMS and polymer mixed with the solvent dyes only allow light within a specific band to pass through. It is important to select an appropriate solvent dye to realize light filtering at a specific cut-off or cut-on wavelength and with a suitable bandwidth to fit the desired fluorescent channels. The solvent dye should be dissolvable in the PDMS and the polymer. To realize light filtering for a certain fluorochrome, the solvent dye should present high absorbance in the excitation band and high transmittance in the emission band of that fluorochrome. Increasing the concentration of the solvent dye in the PDMS and polymer can enhance the absorbance of the light over the full band. Thus, it is necessary to determine a suitable concentration for a reasonable filtering effect, e.g., OD > 4 in the excitation band and OD < 0.5 in the emission band. Lenses in four colours have been developed. The blue lens and the green lens function as bandpass filters with peak wavelengths at 440 and 550 nm, and the yellow lens and the red lens function as longpass filters with cut-on wavelengths at 562 and 594 nm, respectively. Although both the yellow and red lenses exhibit longpass filtering behaviour beyond 594 nm, the red lens is suitable for use with fluorochromes that have an excitation band between 480 and 580 nm, and the yellow lens is favourable for filtering out the light of emission maxima between 560 and 595 nm. The effects of temperature and UV light exposure on the optical and physical properties of the lenses are negligible (for more details, see Figs. S7 and S13 in the Supplementary Information). In addition, contamination (such as dust) on the lens can be easily removed or degreased by using removable tape or rinsing the lens in ethanol.

The colour compound lens can be directly fabricated or flexibly transferred onto any smartphone and combines the functions of optical magnification and light filtering. There is no need to prepare an attachment that needs to be assembled from several elements or that needs to be specifically designed for a certain smartphone model. Since our lenses function irrespective of the model of smartphone, our strategy circumvents the rapid and continuous changes in smartphone technology (see the lens swap amongst four different smartphones in Fig. S14 in the Supplementary Information).

In conclusion, the colour compound lens-based smartphone technology we present here provides a compact, affordable platform for fluorescence microscopy imaging. A smartphone equipped with the lens can capture images with a resolution at the cellular level and an FOV on a tissue-wide scale. Both of these capabilities rely on the pixel and image sensor size within the smartphone; as such, performance could undoubtedly be improved with the emergence of improved smartphone technology. Shih’s group at Houston University invented the three-dimensional printed smartphone microscope lens, named DOTLens, which has already served as a key element for a lightweight smartphone microscope^40–42^. The colour compound lens design presented here could serve as the next generation of multifunctional lens modules for field-portable smartphone microscopes. The outlined qualitative and quantitative bioanalytical demonstrations, including cell and tissue observation, cell counting, and plasmid transfection and superoxide production evaluation, are just the tip of the iceberg for potential applications. Colour compound lenses could be developed for more fluorescent channels, and the capability of the cost-effective microscopic imaging platform could be significantly enhanced. Due to its low cost and simple fabrication process, production of the fluorescence module could be realized in mass quantities to serve as an add-on toolkit for any smartphone used in portable and customized healthcare applications at the point-of-care.

## Materials and methods

### Fabrication of colour lenses

Solvent dye was first dissolved in the PDMS prepolymer (Sylgard 184, Dow Corning, USA) and methyl phenyl polymer (Andisil, AB Specialty Silicones, USA). Sudan IV (Aladdin, China), Sudan II (Aladdin, China), Solvent green 28 (J&I Biological, China), and Solvent blue 59 (Sigma-Aldrich, Germany) were used as red, yellow, green, and blue solvent dyes, respectively. The mixture ratios of the red, yellow, green, and blue solvent in the PDMS prepolymer and the methyl phenyl polymer were 0.42 and 0.45 μg mL^−1^, respectively. PDMS cross-linker was then mixed with the PDMS prepolymer at a weight ratio of 1:5 (the ratio between the cross-linker and the prepolymer). A thin PDMS film was applied on the round protruding camera housing (Model I) and cured for 30 min at room temperature. A lens was then directly formed by dripping the liquid-state PDMS and the polymer on the camera housing. To prevent the PDMS from dropping off the edge of the camera housing and to realize a short focal length, the contact angle, *θ*_PDMS_, must obey the condition *θ*_PDMS_ *<* *θ*_C_. To ensure that this condition is met, the contact angle can be set as the supplementary angle to the subtended angle at the edge, *i.e*., *θ*_PDMS_ *=* *180°–φ*. Using this assumption, the total volume of the PDMS and the polymer that will prevent overflow could then be determined as

1 $$V_{\rm{Total}} = \frac{{\pi D_{\rm{PDMS}}^3}}{{24\left( {\sin \theta _{\rm{PDMS}}} \right)^3}}\left( {2 + \cos \theta _{\rm{PDMS}}} \right)\left( {1 - \cos \theta _{\rm{PDMS}}} \right)^2$$

where *D*_PDMS_ is the diameter of the round protruding camera housing or the disk (Fig. S2 in the Supplementary Information). In addition, the base diameter of the polymer droplet, *D*_Oil_, should be equal to or slightly larger than the aperture of the camera lens. The PDMS was solidified after curing for 8 h at room temperature. The lens formed directly on the camera housing could be easily peeled off for later use or replacement with another preprepared lens. A thin PDMS layer applied to the camera housing, which could be cured after 30 min at room temperature, was used to adhere the spare lens.

If the camera housing was of the other forms (Model II), the lens was fabricated on a glass disk whose diameter fitted the camera housing. A PDMS film was coated onto the glass disk and solidified at 80 °C for 3 min. The PDMS and the polymer were dripped onto the glass disk, successively. The PDMS was solidified after 1 h at 80 °C. Meanwhile, a thin PDMS layer was applied over the camera housing. Finally, the lens was transferred onto the camera housing and could be used after 30 min at room temperature. The lens on the camera housing could be replaced with the spare lens following the procedure in Model I. The models of the smartphones used in the experiments are iPhone 6s Plus and Nokia 7 representing the Model I and Model II camera housings, respectively. For bioanalytical microscopic imaging, the lenses fabricated for the iPhone 6s Plus and Nokia 7 have focal lengths of 4.6 and 5 mm, respectively.

### Measurement of spectral response

Optical spectra of the cured PDMS and polymer solution with and without solvent dye were measured using a spectrophotometer (LAMBDA 1050, PerkinElmer, USA). ODs ranging from 300 to 800 nm were observed, as shown in Fig. S7 in the Supplementary Information. The PDMS and polymer without solvent dye are transparent within the visible spectrum. The PDMS and polymer dyed with the blue solvent dye serve as a bandpass filter with a peak wavelength of ∼440 nm and a −10 dB bandwidth of 50 nm and as a longpass filter with a cut-on wavelength of 680 nm. There is a transmission band centred at 550 nm with a −10 dB bandwidth of 45 nm in the PDMS and polymer dyed with the green solvent dye. If the PDMS and polymer are dyed with the yellow solvent dye and the red solvent dye, they function as longpass filters with cut-on wavelengths of 562 and 594 nm, respectively.

### Quantification of focal length

The focal length of the lens was calculated based on the profile of the PDMS cap and the polymer droplet and verified by experimental measurement. In the theoretical analysis, the PDMS spherical cap can be described as $y_{\mathrm{PDMS}} = \sqrt {\left( {D_{\mathrm{PDMS}}/2\sin \left( {\theta _{\mathrm{PDMS}}} \right)} \right)^2 - x^2} - D_{\mathrm{PDMS}}/2\sin \left( {\theta _{\mathrm{PDMS}}} \right) + h_{\mathrm{PDMS}}$, and the curvature radius of the spherical cap can be expressed as $R_{\mathrm{PDMS}} = D_{\mathrm{PDMS}}/\left( {2\sin \left( {\theta _{\mathrm{PDMS}}} \right)} \right)$, while the polymer droplet is elliptical in shape and the upper surface can be written as $y_{\mathrm{Polymer}} = b\sqrt {1 - x^2/a^2} - b + h_{\mathrm{Polymer}}$, which can be approximated by a quartic polynomial $y_{\mathrm{Polymer}\_\mathrm{Approx}} = - bx^4/8a^4 - bx^2/2a^2 + h_{\mathrm{Polymer}}$ using a Taylor series expansion, where *h*_Polymer_ is the height of the polymer droplet and *a* and *b* are the semi-major and semi-minor axes of the ellipse. The profile of the PDMS cap and the polymer droplet were measured by an optical contact angle meter (SL200B, Kino, USA). Then, the focal length of the lens was obtained using Zemax OpticStudio. In addition, the focal length was also quantified during optical imaging. A checkerboard pattern used as an object was illuminated by an LED light source, and an image of the pattern was formed behind the lens. The distances from the object and the image in focus to the lens are denoted *u* and *v*, respectively (see Fig. S3 in the Supplementary Information). The primary and secondary principal planes of the lens are located at *p*_1_ and *p*_2_. A ray perpendicularly passing through the primary principal plane is refracted at the secondary principal plane. The image distance varies with a change in the object distance. During experimentation, the focal length of the lens can be determined using the paraxial approximation. A group of image distances can first be measured by adjusting the object distances. The focal length and the location of the principal planes can then be calculated based on the following relationship

2 $$\frac{1}{f} = \frac{1}{{u - p_1}} + \frac{1}{{v - p_2}}$$

### Illumination source

An illumination source was developed as shown in Fig. S8 in the Supplementary Information. The size of the source is 100 mm × 88 mm × 55 mm (length × width × height). The sample could be placed on top of the source and illuminated through a 25 mm pupil. A white LED was used for bright-field imaging, and 365, 480, and 520 nm LDs used as excitation light sources for fluorescence imaging were mounted on different chips. Once the LED chip or the LD chip was inserted into the illumination source, the chip was positioned by two tiny magnets and connected to the electrodes, thus turning on the LED or the LD automatically. The source was powered by a 12 V battery. The white LED chip was fixed at left of centre with a tilt angle of 10°, generating oblique illumination. The collimated laser beam illuminated the samples with an incident angle of 45°, which was larger than the acceptance angle of the compound lens. Thus, the excitation light would not be directly coupled into the image sensor, efficiently reducing the background noise during fluorescence imaging.

### Cell culture and preparation

The B16-F0 mouse melanoma cell line, HBEC3-KT human bronchial epithelial cell line, 4T1 mouse breast cancer cell line, 293T human embryonic kidney cell line, A375 human malignant melanoma cell line and B×PC-3 human pancreatic cancer cell line from American Type Culture Collection (ATCC, Manassas, VA, USA), and Huh7 human liver cancer cell line from Riken Bioresource Center, Japan, were cultured in Dulbecco’s modified Eagle medium (DMEM) culture medium supplemented with 10% foetal bovine serum (FBS), 100 U mL^−1^ penicillin and 100 μg mL^−1^ streptomycin. The cells were grown in a 5% carbon dioxide (CO_2_) humidified incubator at 37 °C until 70–80% confluence.

### Preparation of tissue sections

The research study was endorsed by the Ethics Committee of Shanghai Cancer Center, Fudan University (Certificate No. 050432-4-1212B) and the Institutional Animal Care and Use Committee of Shanghai Medical College, Fudan University (Certificate No. 20130227-017). Human liver tissues were sourced from Shanghai Cancer Center, Fudan University. In the tumour transplantation experiment, three nude mice purchased from the Chinese Academy of Science were subcutaneously injected with a suspension of B×PC-3 cells, and after 2 weeks, the tumour tissues were collected from the mice under carbon dioxide euthanasia. The tissues were routinely embedded in paraffin and cut into 3 μm sections. The sections of the human tissue samples were incubated with rabbit anti-human CK18 polyclonal antibody (ThermoFisher Scientific), and the sections of the mouse tissue samples were incubated with rabbit anti-human GAPDH polyclonal antibody (ThermoFisher Scientific) overnight at 4 °C. Then, the sections were incubated with goat anti-rabbit AF488 secondary antibody (ThermoFisher Scientific) for 1 h and DAPI (ThermoFisher Scientific) for 15 min. Finally, the sections were sealed with antifade mountant (ThermoFisher Scientific).

### Plasmid transfection

A total of 500 ng of pEGFP-C2-NLRP3 plasmids (Addgene) were mixed with Lipofectamine 3000 transfection reagent (ThermoFisher Scientific), and the mixture was incubated for 15 min at room temperature to form a complex. Then, the complex was added to 2 × 10^5^ 293T cells. The transfected cells were maintained in DMEM with 10% FBS at 37 °C and 5% CO_2_ for 48 h. Transfection efficiency was determined as the number of 293T cells that expressed EGFP transgene in a total population, which was counted based on DAPI nuclear staining.

### Lipopolysaccharide-induced superoxide production

HBEC3-KT cells were treated with LPS at 50, 100, or 150 μg mL^–1^ for 24 h. MitoSox Red (5 μM) and DAPI (5 μM) were added to the cells. After 15 min of incubation at 37 °C, the cells were detected by the HSFM.

## Supplementary information

Supplementary Information
