# Supplementary material for: Colour compound lenses for a portable fluorescence microscope
Source: Light Sci Appl. 2019 Aug 21;8:75. doi: 10.1038/s41377-019-0187-1 (PMC6804733; doi:10.1038/s41377-019-0187-1)
Supplement: Supplementary file 1 — Supplementary Information [file 41377_2019_187_MOESM1_ESM.docx]

Supplementary Information

**Colour compound lenses for a portable fluorescence microscope**

*Bo Dai^1,†^, Ziao Jiao^1,†^, Lulu Zheng^1,†^, Hunter Bachman^2^, Yongfeng Fu^3^, Xinjun Wan^1^, Yule Zhang^1^, Yu Huang^1^, Xiaodian Han^4^, Chenglong Zhao^5,6^, Tony Jun Huang^2*^, Songlin Zhuang^1^, and Dawei Zhang^1*^*

^1^ Engineering Research Center of Optical Instrument and System, the Ministry of Education, Shanghai Key Laboratory of Modern Optical System, University of Shanghai for Science and Technology, Shanghai, 200093, China

^2^ Department of Mechanical Engineering and Materials Science, Duke University, Durham, NC 27709, USA

^3^ Department of Medical Microbiology and Parasitology, School of Basic Medical Sciences, Fudan University, Shanghai, 200032, China

^4^ Department of Laboratory Medicine, Shanghai Cancer Center, Fudan University, Shanghai, 200032, China

^5^ Department of Physics, University of Dayton, Dayton, Ohio, 45469, USA

^6^ Department of Electro-Optics and Photonics, University of Dayton, Dayton, Ohio, 45469, USA

**^†^** These authors contributed equally: Bo Dai, Ziao Jiao, Lulu Zheng

* Correspondence and requests for materials should be addressed to D.Z. (email: [dwzhang@usst.edu.cn](mailto:dwzhang@usst.edu.cn), Phone: +86 55272096) or T.J.H. (email: [tony.huang@duke.edu](mailto:tony.huang@duke.edu), Phone: (919) 684-5728)

**Supplementary Figures**


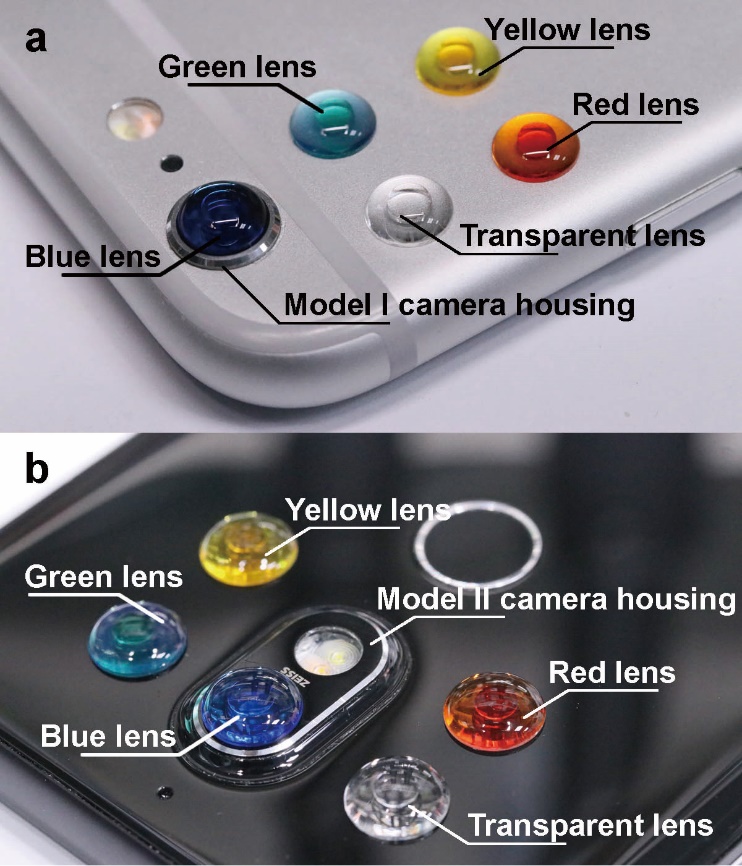


**Figure S1: The lenses fabricated for different models of smartphones. a)** Lenses that are directly fabricated onto the smartphone with a Model I camera housing. Transparent, red, yellow and green lenses have been peeled off from the camera housing, and a blue lens remains on the camera. **b)** Lenses fabricated on a glass disks. The blue lens has been transplanted onto the camera housing, and the remaining lenses are for different fluorescent channels.


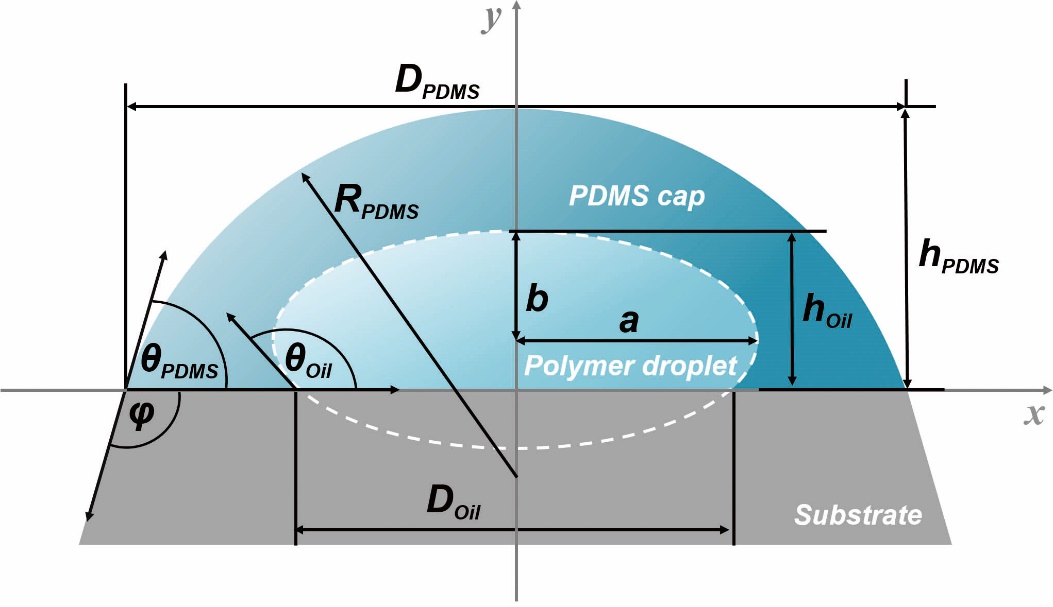


**Figure S2: Model of the compound lens.** The PDMS cap and the polymer droplet have axisymmetric profiles. The surface of the PDMS spherical cap can be expressed as $y_{PDMS}=\sqrt{\left( D_{PDMS}/2\sin\left( \theta_{PDMS} \right) \right)^{2}-x^{2}}-D_{PDMS}/2\sin\left( \theta_{PDMS} \right)+h_{PDMS}$, while the interface of the PDMS and the polymer droplet can be approximated to a quartic polynomial, i.e. $y_{Polymer\_Approx}=-bx^{4}/8a^{4}-bx^{2}/2a^{2}+h_{Polymer}$.


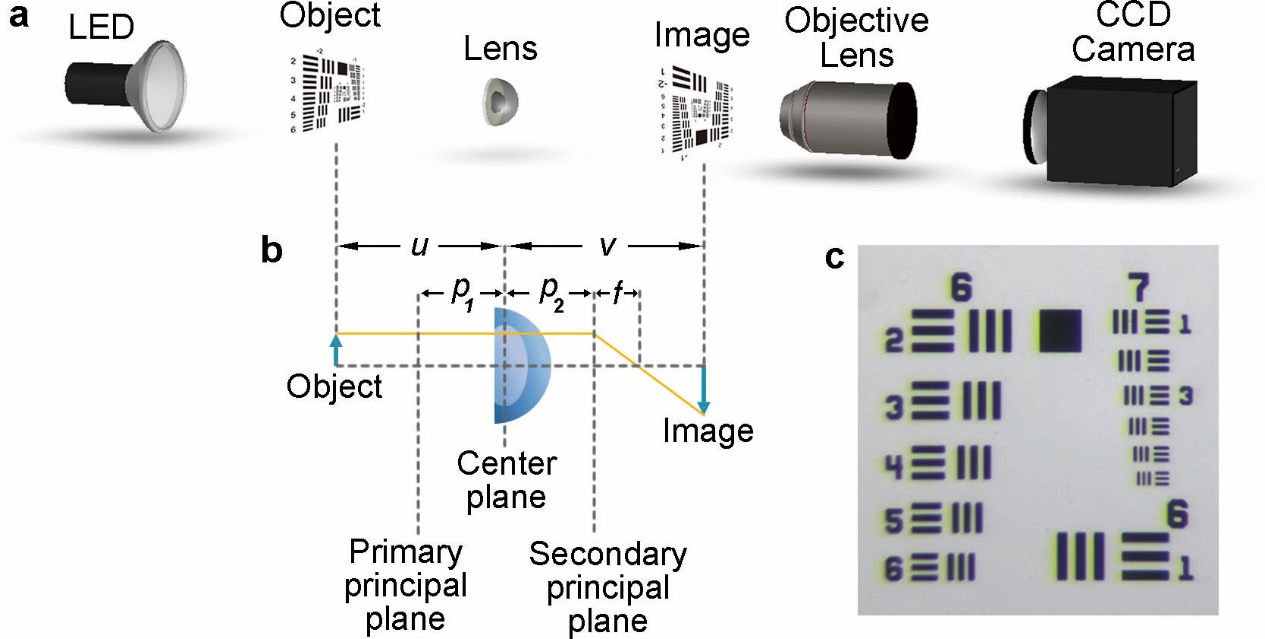


**Figure S3: Quantification of the focal length. a)** Optical setup for measuring the focal length. **b)** Ray diagram for the compound lens. By varying the distance of the object, i.e. *u*, the image distance, i.e. *v*, is measured. Then, the focal length can be determined based on $1/f=1/\left( u-p_{1} \right)+1/\left( v-p_{2} \right)$. **c)** Image projected by the lens and captured by the CCD camera.


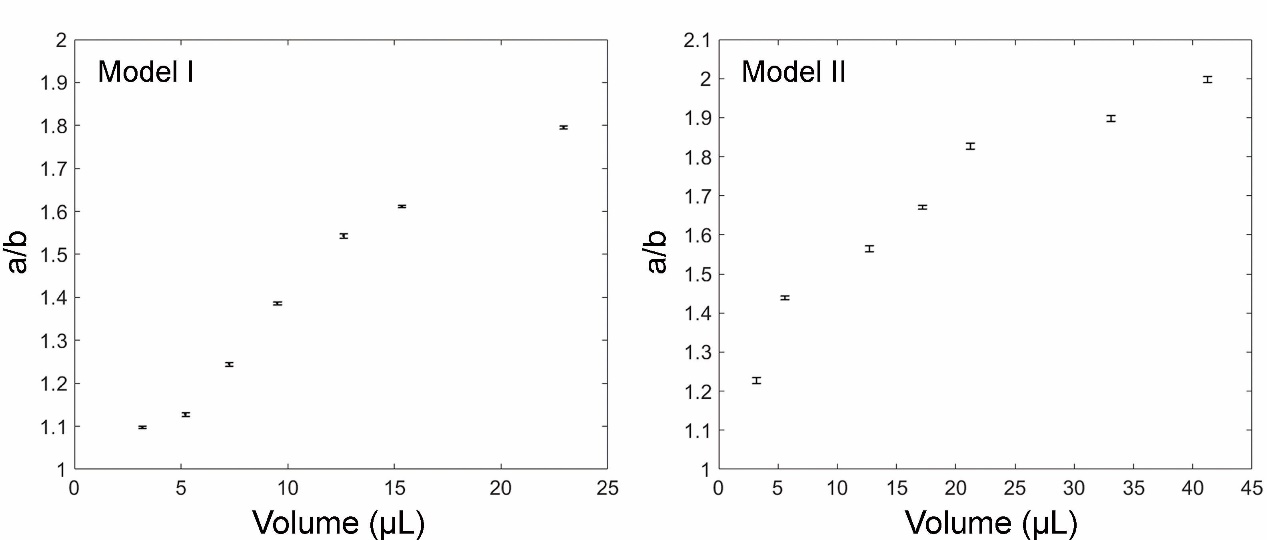


**Figure S4: The ratio between the semi-major and semi-minor axes of the elliptical polymer droplet.** The increase in the ratio is attributed to the gravity effect and indicates that the curvature of the upper surface of the polymer droplet is reduced.


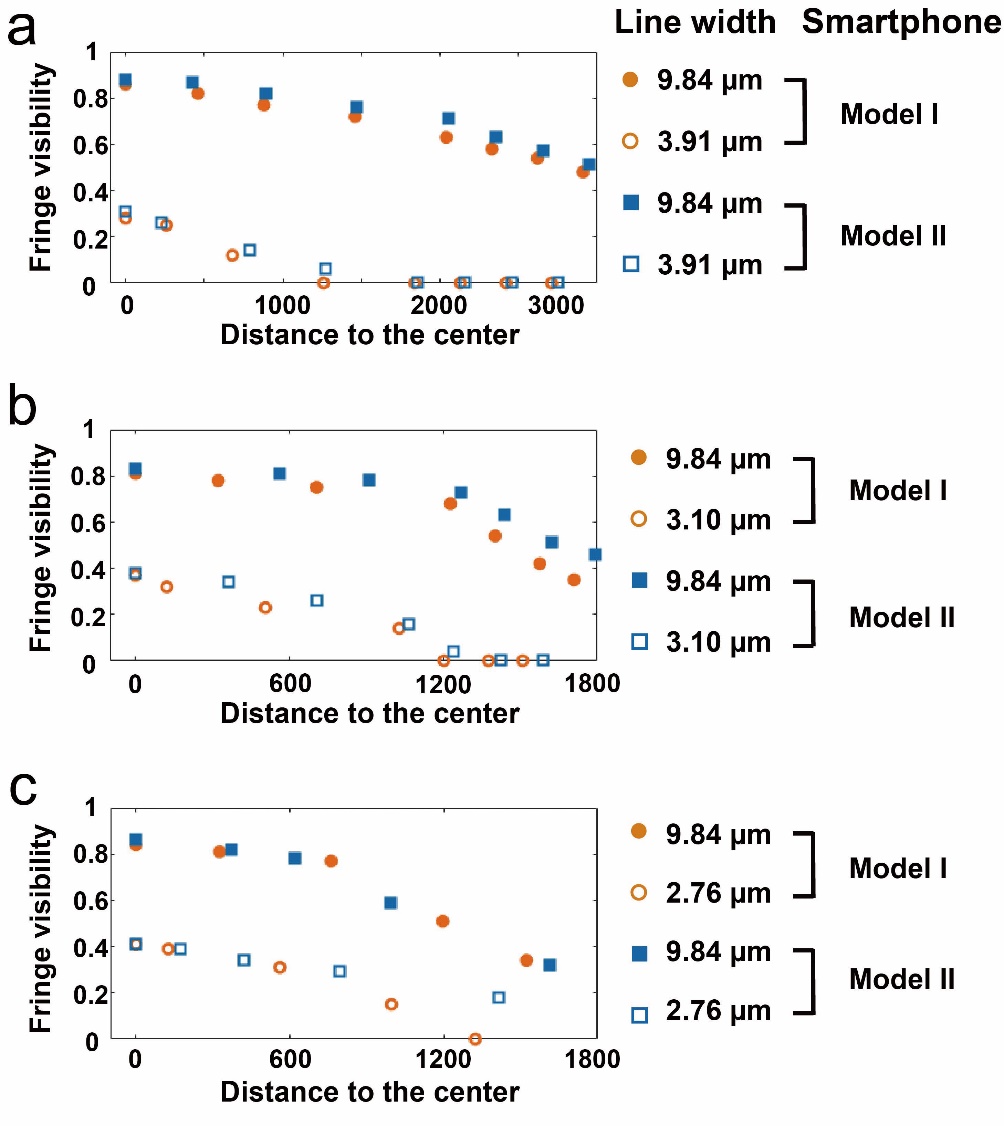


**Figure S5: Evaluation of curvature-field aberration.** The lines on the resolution target USAF-1951 are moved from the center to the edge of the view. The images of the lines are captured by the Model I smartphone equipped with the lenses of **a)** 7.2 mm, **b)** 5.7 mm and **c)** 4.6 mm focal length and the Model II smartphone equipped with the lenses of **a)** 7.9 mm, **b)** 6.8 mm and **c)** 5.1 mm focal length. The fringe visibility is defined as *(I_max_–I_min_)/(I_max_+I_min_)*, where *I_max_* is the brightness of the lines and *I_min_* is the brightness of the space in between the two adjacent lines. The fields with a fringe visibility above 0.3 can be regarded as the effective FOV.


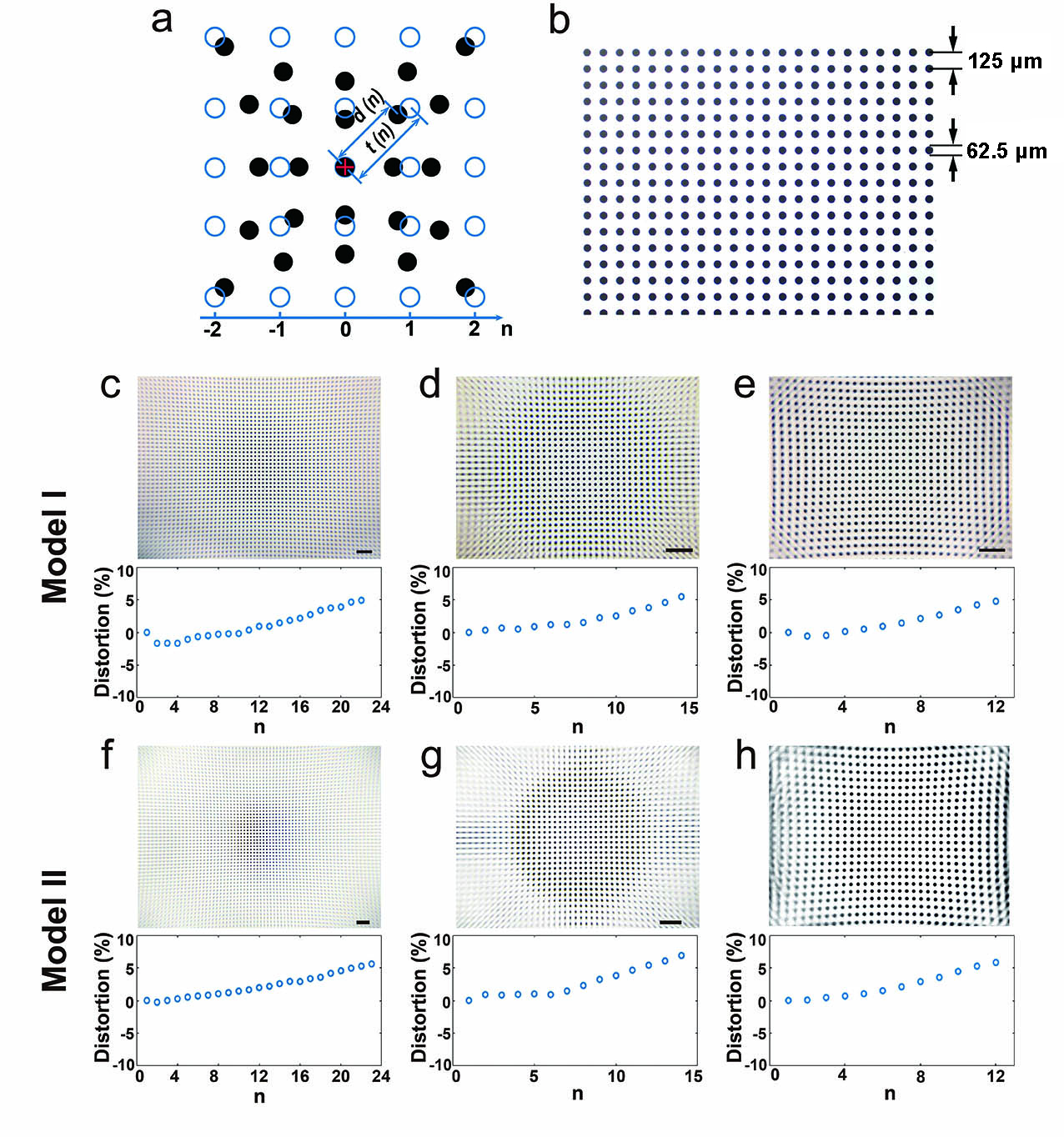


**Figure S6: Evaluation of Pincushion distortion. a)** An illustration of the fixed frequency grid (blue circles) and the image with pincushion distortion (black dots). The red cross is the center of the image. *t(n)* represents the theoretical distance from the center to the dot and *d(n)* is the actual distance in the image from the center to the dot, where *n* represents the *n*th dot from the center diagonally. **b)** The microscopic image of the fixed frequency grid distortion target captured by a conventional microscope (Axio Vert.A1, Carl Zeiss, Germany) equipped with a 5× objective lens (EC Epiplan, Carl Zeiss, Germany). **c)-e)** The images captured by the Model I smartphone equipped with the lenses with focal lengths of 7.2 mm, 5.7 mm and 4.6 mm. **f)-h)** The images captured by the Model II smartphone equipped with lenses with the focal lengths of 7.9 mm, 6.8 mm and 5.1 mm. The Pincushion distortion is defined as *Distortion=(t(n)–d(n))/t(n)×100%*. Since ±4% distortion is just noticeable to human eyes, whereas ±10% distortion is definitely objectionable, the field with the distortion below 4% could be regarded as the effective FOV. Scale bars: 500 μm.


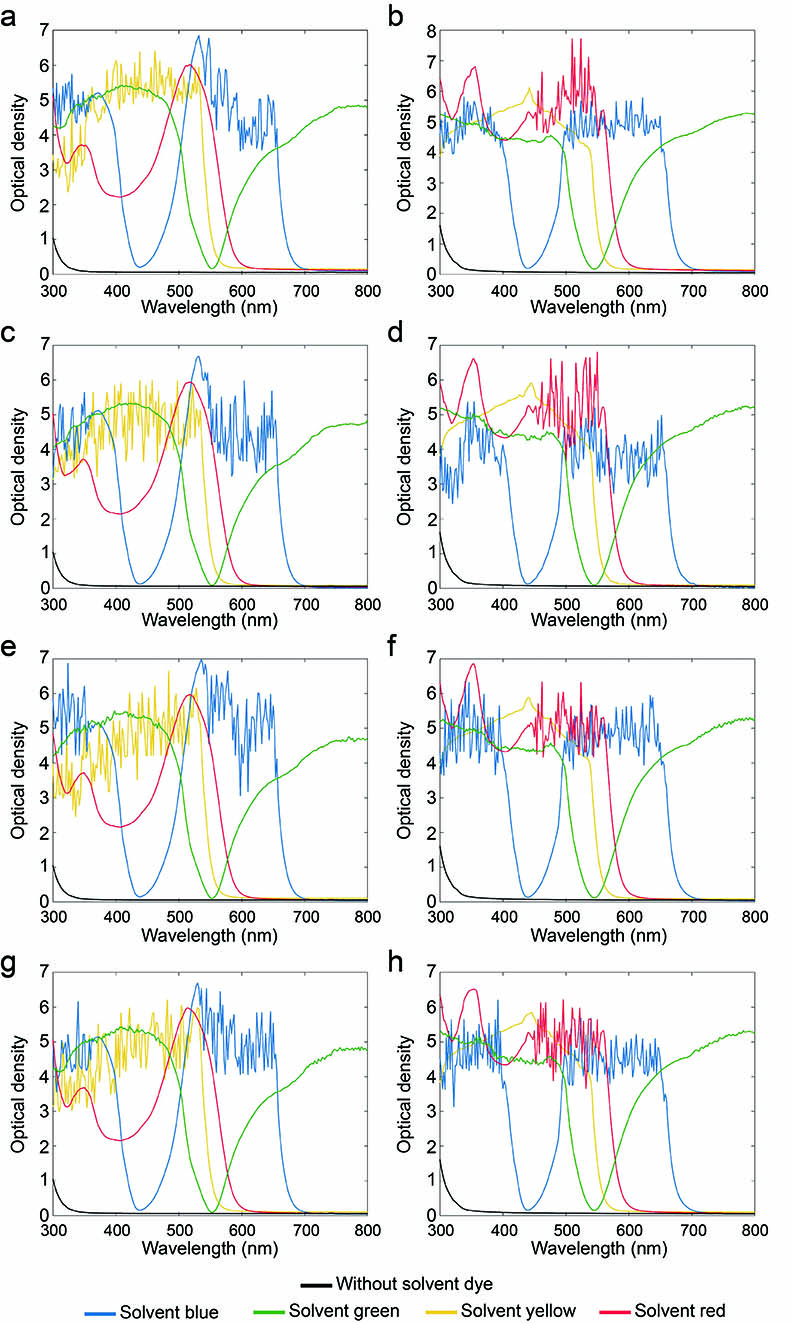


**Figure S7: Spectral response of the cured PDMS and the methyl phenyl polymer with and without the solvent dye. a)** OD of the cured PDMS mixed with and without the solvent dye at the concentration of 0.42 μg mL^–1^. **b)** OD of the methyl phenyl polymer mixed with and without the solvent dye at the concentration of 0.45 μg mL^–1^. **c)** and **d)** OD of the PDMS and the methyl phenyl polymer after heating at 40 ºC for 6 hours. **e)** and **f)** OD of the PDMS and the methyl phenyl polymer after freezing at –5 ºC for 6 hours. **g)** and **h)** OD of the PDMS and the methyl phenyl polymer after the exposure under the 3 Watt 365 nm UV LED for 1 hour.


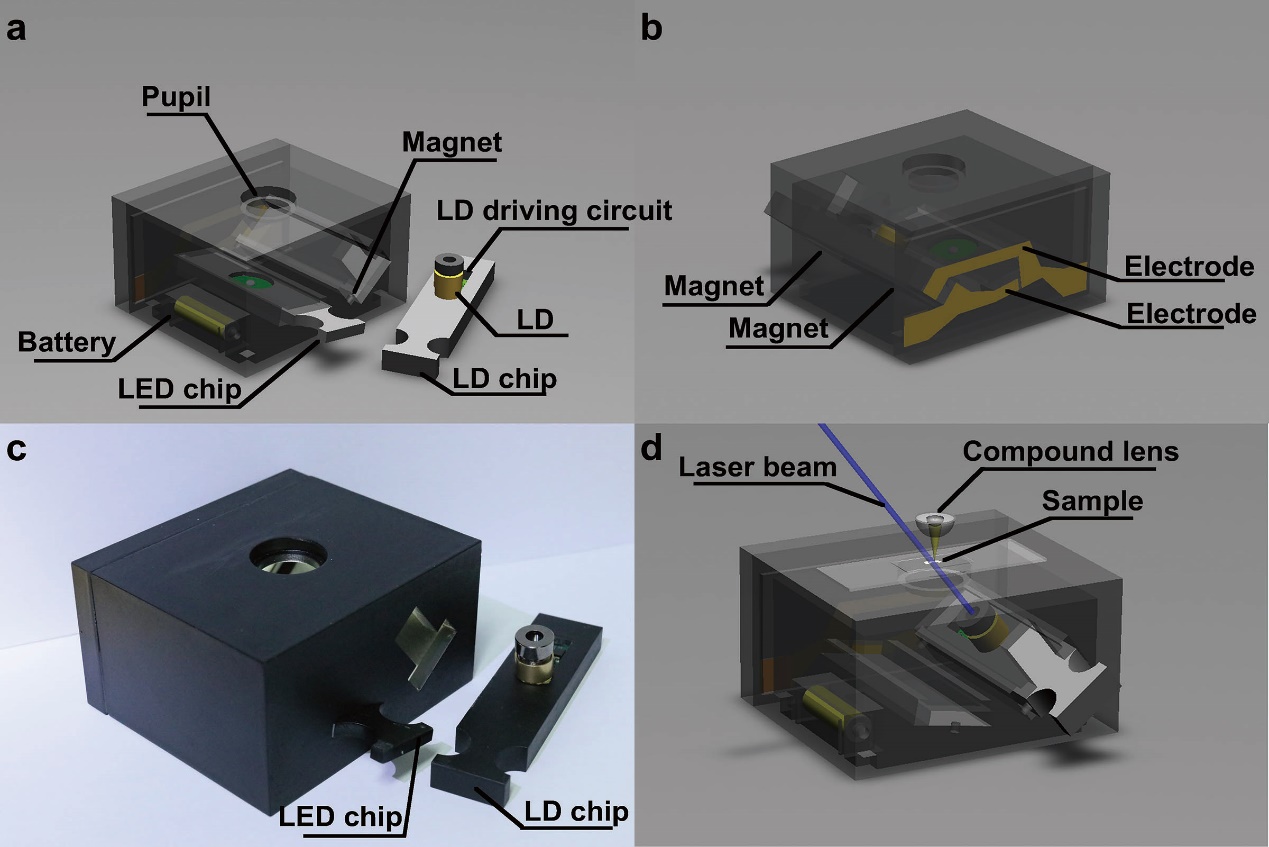


**Figure S8: 3D schematic diagrams and photograph of the illumination source. a)** Front view of the illumination source. There are two slots for the installation of the white-light LED chip and the LD chips for 365 nm, 480 nm and 520 nm light which can be fixed by two tiny magnets. The source is driven by a 12 V battery. A pupil is on the top of the source. **b)** Back view of the illumination source. The electrodes are on the back of the source. Once the LED chip or the LD chip snaps into place, the chip connects to the electrodes automatically and the light is switched on. **c)** Photograph of the illumination source. The LED chip is inserted into the source and the LD chip is beside the source. **d)** The laser beam has larger incident angle comparing to the acceptance angle of the compound lens, efficiently reducing the background noise in the fluorescence imaging.

**
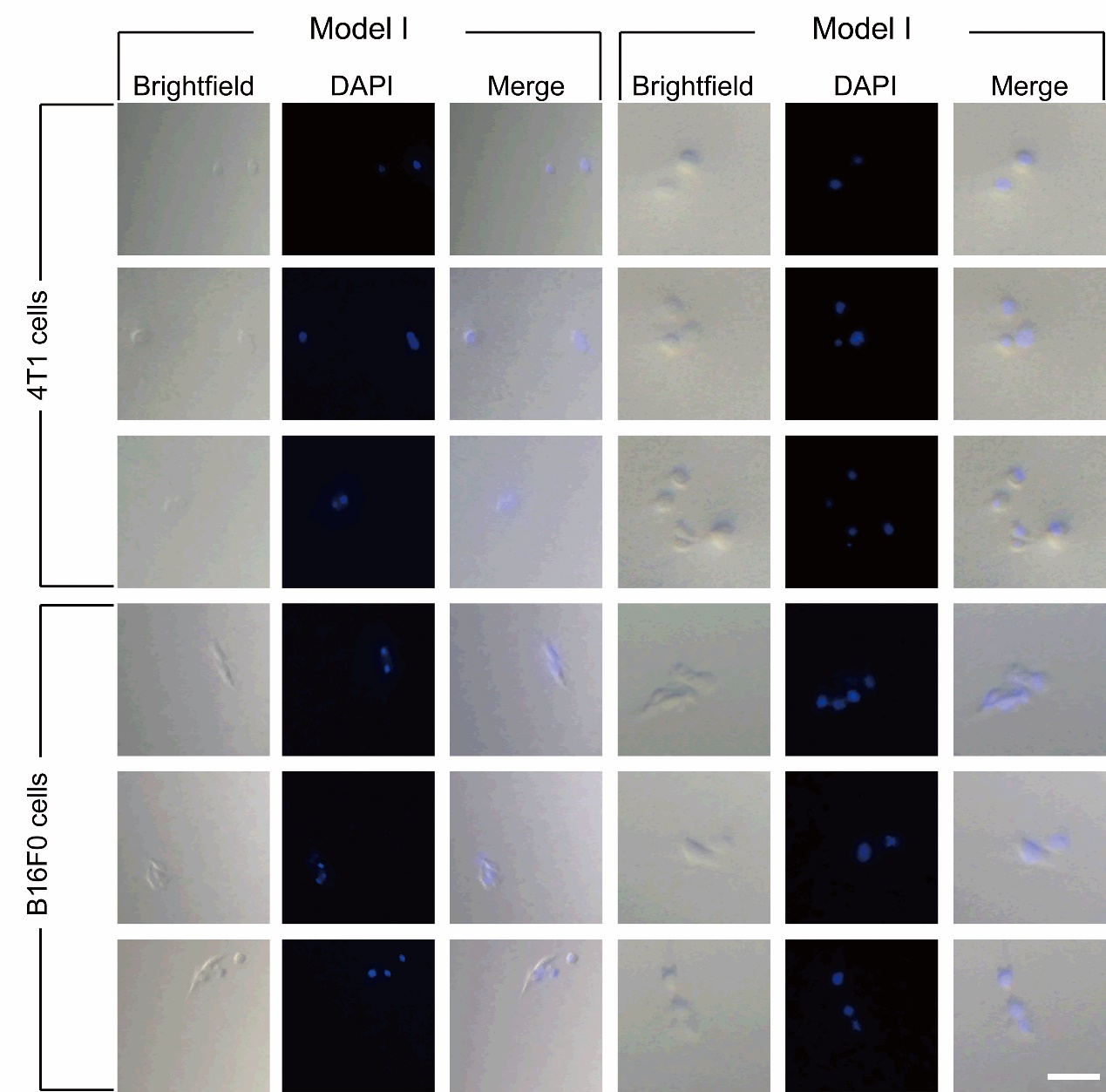
**

**Figure S9: Bright-field and fluorescence images of the cells stained with DAPI using the HSFM.** The smartphone equipped with the transparent lens and the blue lens is used to capture the bright-field and fluorescence images under white light and 365 nm UV light respectively. The lenses have focal length of 4.6 mm (Model I) and 5 mm (Model II). The ISO sensitivity of the camera is set at 50 for bright-field images and 800 for fluorescence images. The shutter speed is 1/50 second (Model I) and 1/60 second (Model II) for bright-field imaging and 1/5 second (Model I) and 1/10 second (Model II) for fluorescence imaging, respectively. White balance is fixed to 4500 K. The cells are stained with DAPI for 15 minutes before the imaging. Scale bars = 50 μm.


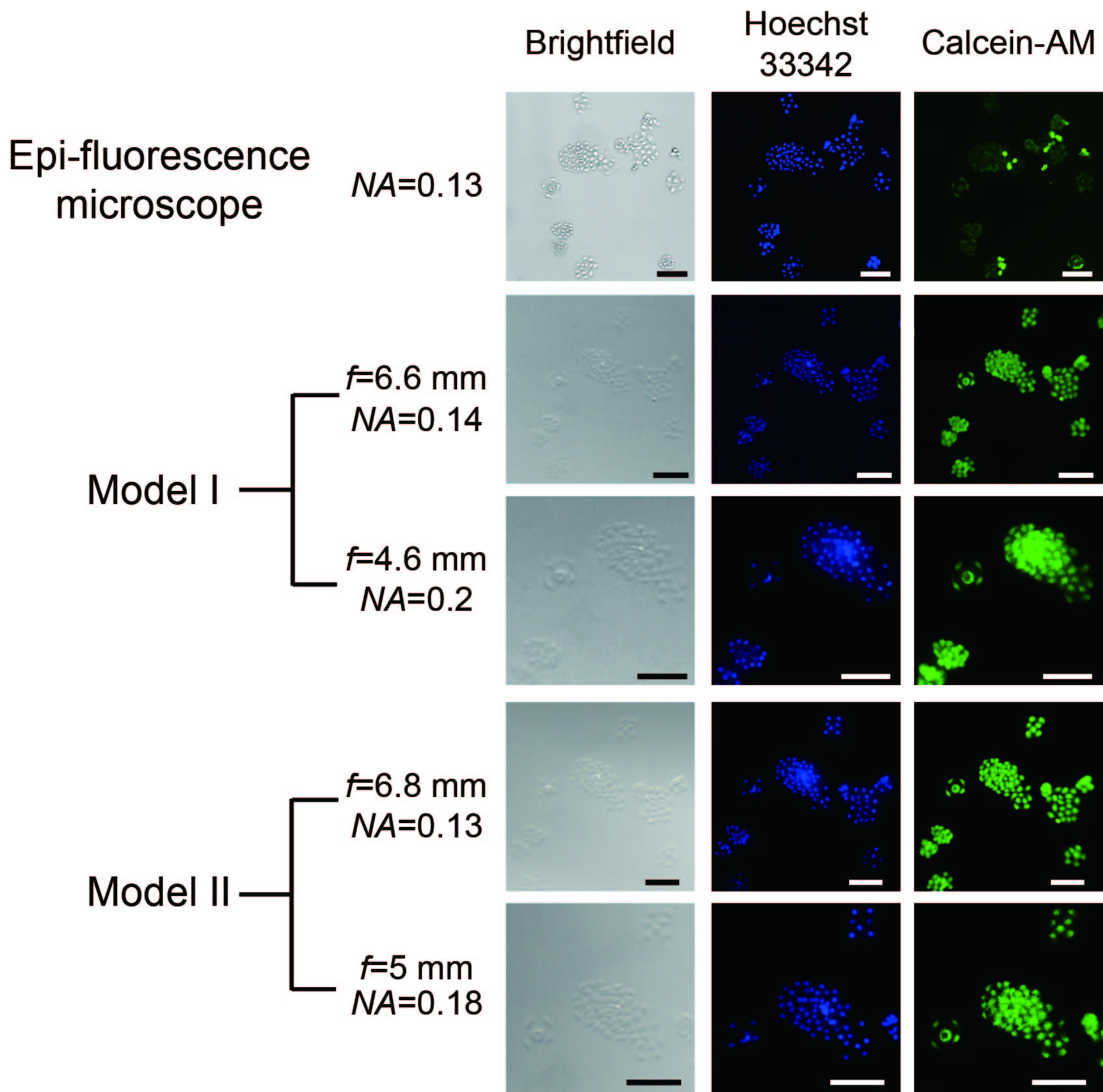


**Figure S10: Microscopic images of 4T1 cells.** 4T1 cells are co-stained with Hoechst 33342 and Calcein acetoxymethyl ester (AM) for 30 minutes at 37 ºC. Hoechst 33342 is a nucleic acid dye for live cells with excitation and emission maxima of 350 nm and 450 nm, respectively. Intracellular esterase of live cells could cleave AM group of cell membrane-permeable Calcein AM, generating membrane-impermeable Calcein which can emit green light when excited by a light around 490 nm. Bright-field and fluorescence images are captured by an epi-fluorescence microscope (IX73 inverted microscope, Olympus, Japan) equipped with a 4× objective lens (UPLFLN, Olympus, Japan) and the smartphones equipped with the transparent, blue and green color compound lenses under white light, 365 nm UV light and 480 nm light respectively. Scale bars = 50 μm.


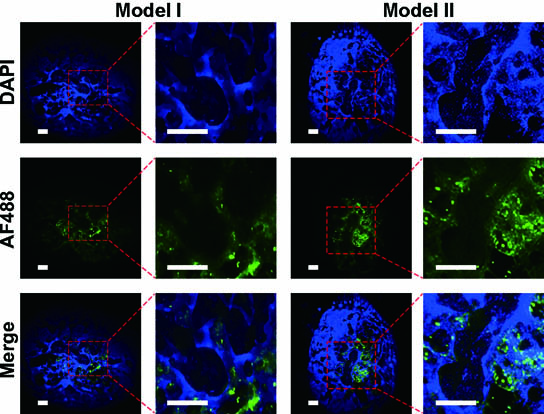


**Figure S11: Fluorescence images of a BxPC-3 tumor xenograft tissue using the HSFM.** The tissue was incubated with the rabbit anti-human GAPDH polyclonal antibody and stained with DAPI and AF488. The large-view and zoom-in images were captured by the smartphone equipped with the blue lens and the green lens. Scale bar = 300 μm.


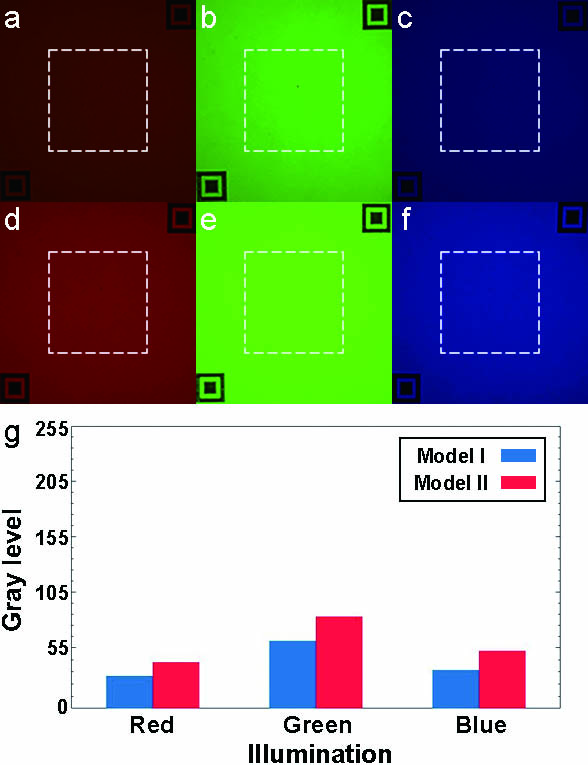


**Figure S12: Sensitivity of the image sensors.** The sensitivity of the camera was tested under the illumination in red, green and blue. The illumination was generated by an array of three-color LEDs via a diffuser. The illuminance of the red, green and blue light was 16.6 lx, 173.6 lx and 152.3 lx respectively. The homocentric-square markers on the corners were used for focusing. The cameras had a shutter speed of 1/30 second at 100 ISO sensitivity. **a)-c)** The images captured by the Model I smartphone. **d)-f)** The images captured by the Model II smartphone. **g)** The average values in gray scale were calculated from the images within the dashed squares. Model I: iPhone 6s Plus smartphone. Model II: Nokia 7 smartphone.

**
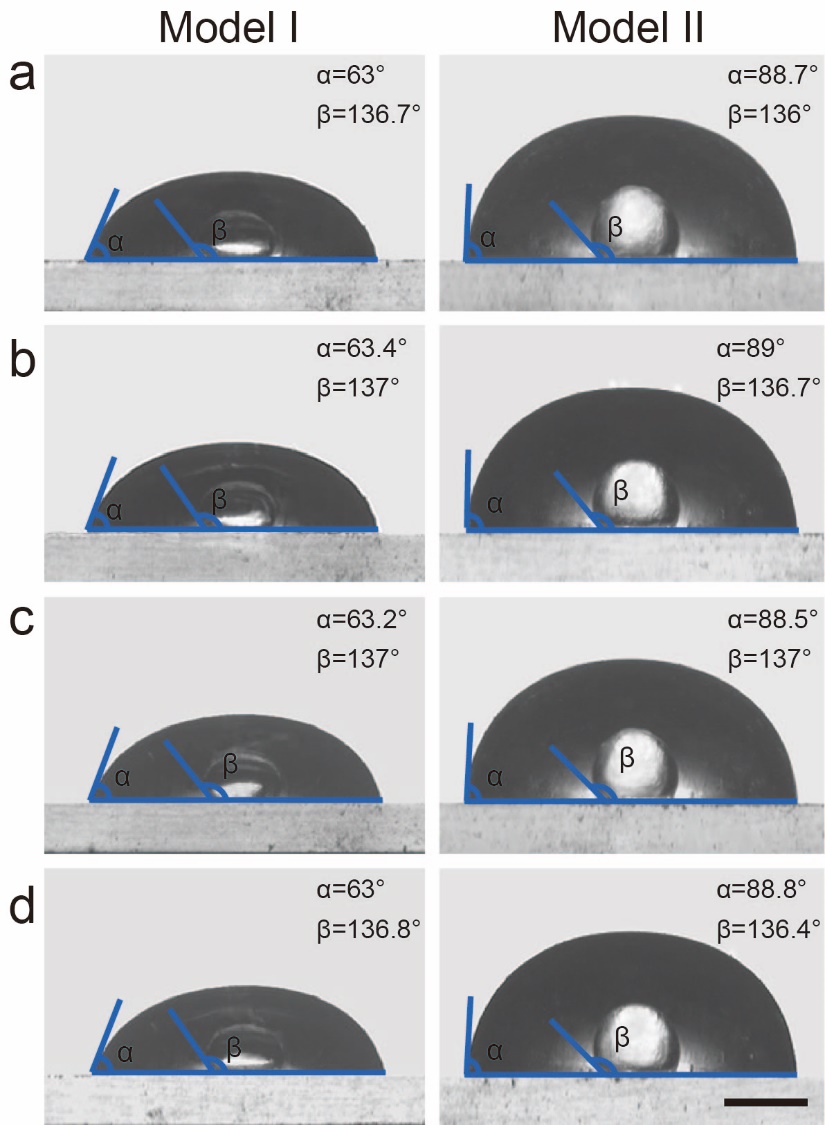
**

**Figure S13: The influence of natural factors on the shape of the lens. a)** The lenses to be tested under extreme conditions; lenses were fabricated with a polymer volume of 3.5 μL. **b)** The lenses after heating at 40 ºC for 6 hours. **c)** The lenses after freezing at –5 ºC for 6 hours. **d)** The lenses after the UV exposure under the 3 Watt 365 nm UV LED for 1 hour. Properties of the lenses remained unchanged after each testing procedure, indicating their resistance to wear.


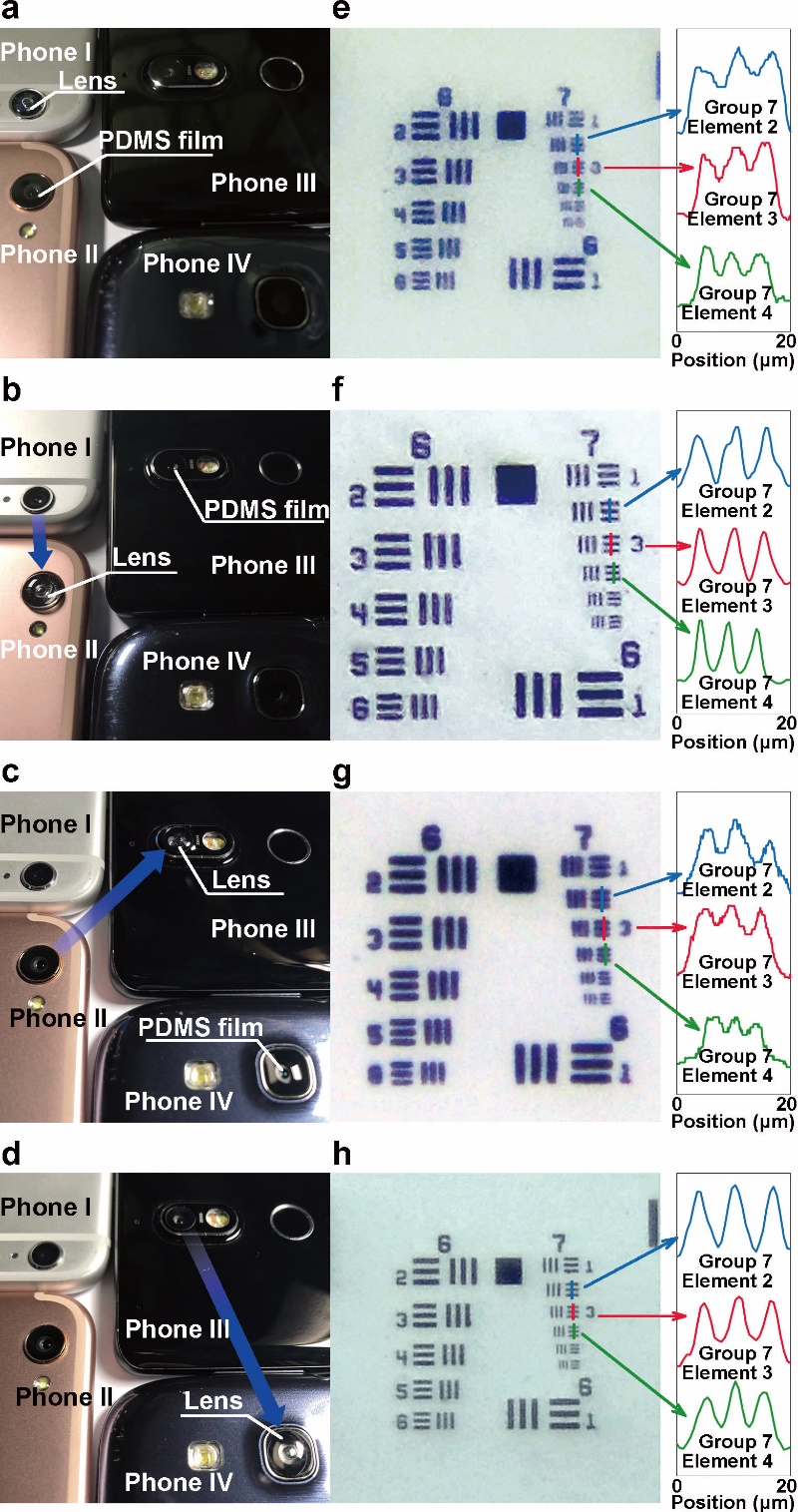


**Figure S14: The compound lens swapped between different smartphones and a comparison of their imaging capabilities. a)-d)** The lens can be easily swapped from one smartphone to another; the same lens was transplanted between phones of a similar camera housing model to capture these photos. The models of the smartphones used in the experiments were: iPhone 6s Plus (Phone I), VIVO X9s (Phone II), Nokia 7 (Phone III) and Samsung Galaxy S3 (Phone IV). Phone I and Phone II represent Model I camera housing, whereas Phone III and Phone IV represent Model II camera housing. **e)-h)** Images of the resolution target USAF-1951 captured by the smartphones with the highest camera zoom (Phone I: 5× camera zoom, Phone II: 8× camera zoom, Phone III: 8× camera zoom and Phone IV: 4× camera zoom). Right insets show the intensity profiles along the blue, red and green lines.

**
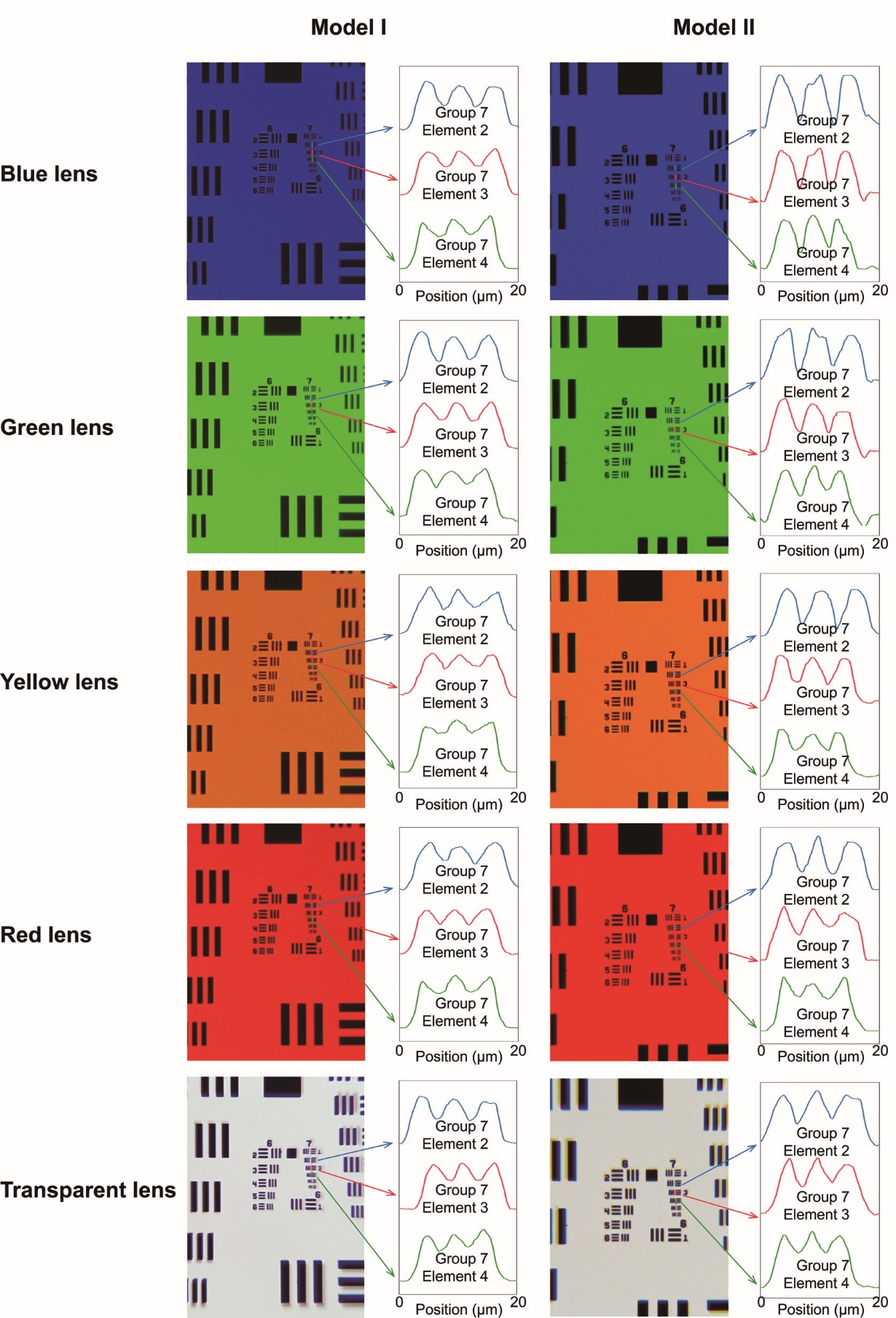
**

**Figure S15: Images captured from the same spot of the resolution target using the smartphone equipped with different color lenses.** Right insets show the intensity profiles along the blue, red and green lines. The polymer droplets inside the lenses have a volume of 3.5 μL.

**Supplementary Note: Image registration**

During fluorescence microscopic imaging, fluorescence channels can be shifted easily by changing the color lenses. Since the uniformity in shape of different color lenses can be guaranteed, the images captured by the lenses have almost no difference in resolution, as shown in Fig. S15. During the imaging, a tripod that mounts the smartphone can be used for image stabilization and focus adjustment. To capture images at the same spot of a sample, it is necessary to first observe the sample under white-light illumination and record the target as a reference image. Once the fluorescence channel has been shifted, the reference spot found under the white-light illumination can be identified. After all of the images are acquired, image registration can be applied to precisely align the set of images. The geometrical transform between the images captured under the white-light illumination and the reference image can be calculated and used to align the images captured from the different fluorescence channels into the same coordinate. An example for bright-field image and fluorescence imaging in the different fluorescence channels based on this procedure is demonstrated in Fig. S10.
